# Predicting GP visits: A multinomial logistic regression investigating GP visits amongst a cohort of UK patients living with Myalgic encephalomyelitis

**DOI:** 10.1186/s12875-020-01160-7

**Published:** 2020-06-10

**Authors:** R. Stephen Walsh, Andrew Denovan, Kenneth Drinkwater, Sean Reddington, Neil Dagnall

**Affiliations:** Manchester Metropolitan University, Manchester, UK

**Keywords:** Myalgic Myalgic encephalomyelitis (ME), Chronic fatigue syndrome (CFS), General practitioner (GP) visits

## Abstract

**Background:**

Myalgic Encephalomyelitis (ME) is a chronic condition whose status within medicine is the subject of on-going debate. Some medical professionals regard it as a contentious illness. Others report a lack of confidence with diagnosis and management of the condition. The genesis of this paper was a complaint, made by an ME patient, about their treatment by a general practitioner. In response to the complaint, Healthwatch Trafford ran a patient experience-gathering project.

**Method:**

Data was collected from 476 participants (411 women and 65 men), living with ME from across the UK. Multinomial logistic regression investigated the predictive utility of length of time with ME; geographic location (i.e. Manchester vs. rest of UK); trust in GP; whether the patient had received a formal diagnosis; time taken to diagnosis; and gender. The outcome variable was number of GP visits per year.

**Results:**

All variables, with the exception of whether the patient had received a formal diagnosis, were significant predictors.

**Conclusions:**

Relationships between ME patients and their GPs are discussed and argued to be key to the effective delivery of care to this patient cohort. Identifying potential barriers to doctor patient interactions in the context of ME is crucial.

## Background

Healthwatch is an independent national champion for people who use health and social care services in the UK. In 2015, Healthwatch Trafford received a complaint from a person who identified as having Myalgic Encephalomyelitis (ME) concerning treatment from their GP (general practitioner). The complaint opined that others with ME shared similar negative experiences. Specifically, a lack of patience with ME on the part of healthcare providers, and a lack of knowledge of ME. In response, Healthwatch Trafford ran a patient experience-gathering project, by creating a detailed survey, designed to investigate, how key factors (i.e., length of time with ME, geographic location, trust in GP and gender) impacted upon individual patient relationships with their doctor.

ME as a Condition.

ME is a multisystem condition characterised by fatigue that endures for at least 6 months and remains unrelieved by rest [1–3], it is often debilitating and produces significant functional impairment [4]. According to the Centres for Disease Control and Prevention (CDC) criteria [5] diagnosis of ME or CFS (chronic fatigue syndrome) as it also known, is dependent on the presence of at least four of a range of neuropsychiatric and rheumatologic symptoms [2, 5, 6]. These include impaired short-term memory and concentration; headaches, sensory disturbances; unrefreshing sleep; muscle weakness; tender cervical or axillary lymph nodes; odynophagia, gastrointestinal illness, intolerance to extreme temperatures; and arthralgia [5, 7, 8]. Collectively, symptoms characterise serious functional disorder [6].

It is important to note that there is an ongoing debate about key ME symptoms. Subsequently, different diagnostic criteria exist. For example, De Gucht, et al. [9] acknowledged the presence of mental fatigue, yet no additional somatic symptoms.

Symptoms vary also as a function of age and gender [6, 8]. Collin et al. [10] reported that adolescents, compared to adults, were less likely to have anxiety and more likely to display comorbid depression. Recent research [11–13] indicates that ME is a function of widespread inflammation and multi-systematic neuropathology. As such, there is something of a consensus that, because the term ‘Myalgic Encephalomyelitis’ (ME) indicates underlying pathophysiology, it is appropriate to refer to the disease as ME rather than CFS (chronic fatigue syndrome) [11, 14].

Due to the diagnostic issues alluded to above, some GPs regard ME as a contentious illness, while others report a lack confidence with diagnosis and condition management [4, 15, 16]. A lack of certainty with regard to how medical practitioners might refer patients to specialist services is a further issue reported in the literature [3, 17]. In terms of prevalence, because there is a lack of epidemiological data within the UK estimated ME incidence derives from trends within other countries. Based on these data, ME incidence is at least 0.2–0.4% [8]. In the UK this represents 1 in 250 of the population or 260,000 in total affected [12].

Studies with broader screening procedures report higher rates 0.2–6.4% [13]. Additional estimated frequency varies as a function of diagnostic criteria used (3). Within suffers there is higher incidence of ME in women and young adults (6). Although patients often report alleviated symptoms, full recovery rates are low [7, 14, 15]. Due to symptom complexity and ongoing issues, ME diagnosis and treatment are health care resource intense [18, 19]. Additionally, ME produces major socio-economic costs related to functional impairment and the inability to work [17, 20]. Collectively, direct (e.g., medication, complimentary treatments and primary and secondary care contacts) and indirect factors (welfare payments, losses in work productivity, etc.) are financially and socially expensive [3, 16, 20]. Hunter et al. [12] estimated that the true total cost to the UK economy of ME in 2014/15 was between 1.7 and 4.8 billion pounds.

Central to the diagnosis and treatment of ME is the patient’s relationship with their GP [4, 18]. However, ‘unhelpful attitudes and ignorance are still widespread in primary care’, with levels of acceptance and knowledge of ME amongst GPs reported as often being unsatisfactory [19]. Nevertheless, an important factor effecting condition management and outcome is GP visits [13, 20, 21]. Interestingly, multidisciplinary research (e.g., epidemiology, sociology, and psychology) has highlighted that GP visits are complex situations, in that there is a link between an individual’s social relationships, level of GP interaction (in and out of surgery) and overall health [19, 22].

In particular, Williams et al. [23] proposed that the groups individuals belong to determine symptom appraisals and responses, health related norms and behaviours, coping, social support and clinical outcomes. This applies to both sides of the doctor-patient interaction.

For instance, Saunders [17] reported that many GP’s found that a feeling of belonging to a group ‘suffering with CFS/ME’ is extremely beneficial for many patients. This sense of affiliation provides an understanding and shared ownership which enables the GP and patient to partake on a trustworthy, honest and interactive journey. Alternatively, where doctor’s do not acknowledge, or know, about living with ME, patients may experience higher levels of depression, anxiety, and social exclusion [18, 21, 24].

National Institute of Health and Care Excellence [12] guidelines state that patients should receive a treatment plan tailored to their symptoms. This guidance derives from discussion and appraisal of treatment risks and benefits. Therefore, failure to visit GPs has a potentially detrimental effect on patient condition management and outcome. As such, the aim this paper was to examine factors that might influence patient GP visits (e.g., time with ME, geographic location, trust in GP, formal diagnosis, time to diagnosis and gender) amongst a group of participants who are living with the condition.

## Method

### Source of data

Following receipt of the complaint outlined in the opening paragraph, Trafford Healthwatch undertook preliminary investigations that suggested that, anecdotally at least, complaints about GP attitudes to ME patients were relatively common. Commencing in April 2015, the online tool Survey Monkey was used to collect information pertaining to ME patient experiences. The data used in this analysis is available via the MMU (Manchester Metropolitan University) repository.

### Ethics consent and permissions

In 2017, Trafford Healthwatch contacted MMU to conduct an examination of these anonymised data. Prior to analysis, MMU, Health Psychology & Social Care research ethics committee provided ethical approval. (Ethics Checklist no 1564 18/01/2018). When collecting the (anonymous) online survey data Trafford Healthwatch followed the convention whereby, the researchers having no direct contact with participants, participants by their action of completing the survey imply consent.

### Participants

In total 476 (411 women and 65 men) individuals with ME took part in this study, with 463 participants reporting a formal diagnosis of ME. Due to the close vicinity of the authors to Greater Manchester and the specific interest to ME health services in this area, fifty-nine respondents were from Greater Manchester. To ensure generalizability to the ME population in the UK [21, 24] 417 participants were from the rest of the UK.

### Predictors

The predictor variables were length of time with ME, whether a patient resided in a Greater Manchester borough or the rest of the UK (location), trust in the GP, whether a patient had received a formal diagnosis for ME (formal diagnosis), the time to diagnosis of ME, and gender. All predictor variables were categorical (see Table 1 for information on specific categories).

**Table 1:** Categorical characteristics of the sample

Variable	Category	Number	% of the sample
Time with ME	1–2 years	32	6.7
	2–5 years	68	14.3
	5–10 years	120	25.2
	10–15 years	81	17
	15–20 years	78	16.4
	> 20 years	97	20.4
Location	Rest of UK	417	87.6
	Manchester borough	59	12.4
Trust in GP	Yes	119	25
	No	224	47.1
	Unsure	133	27.9
Formal diagnosis	Yes	463	97.3
	No	13	2.7
Time to diagnosis	< 3 months	28	5.9
	3–6 months	76	16
	6 mths-1 year	119	25
	1–2 years	77	16.2
	2–5 years	78	16.4
	> 5 years	98	20.6
Gender	Female	411	86.3
	Male	65	13.7
Number of GP visits	1–2 times a year	134	28.2
	3–4 times a year	161	33.8
	5–6 times a year	113	23.7
	Monthly	68	14.3

### Outcome

The outcome variable was number of GP visits per year (i.e. 1–2; 3–4; 5–6; monthly).

### Sample size

According to Van Smeden et al. [25] a minimum of 30 observations per independent variable is necessary to achieve empirical validity when conducting multinomial logistic regression. Therefore, a minimum sample of 180 provides a sufficient number of observations for testing the predictive model. This study used a sample of 476 participants, well above the minimum requirement.

### Missing data

The survey incorporated a forced response option. This ensured that participants could not progress through the survey without completing the previous questions. This method ensured the dataset did not contain random instances of missing data. Within the sample, 32 participants failed to complete the survey, indicating an initial sample of 508. Exclusion of these 32 participants occurred prior to calculation of sample size and data analysis.

### Statistical analysis

To meet the study objectives a multinomial logistic regression was undertaken. For this analysis, the sample split into four groups based on how frequently participants visited a GP (1–2 times a year, 3–4 times a year, 5–6 times a year, monthly).

## Results

Multinomial logistic regression requires a careful assessment of univariate and multivariate outliers, multicollinearity, and distribution of the error terms. All standardised values were above − 3.29 and below 3.29, indicating no univariate outliers [26]. The values for Cook’s Distance were less than 1 (specifically .046) suggesting no multivariate outliers. Multicollinearity was not an issue, as VIF <  3 and Tolerance > .1. The P-P plot revealed the error terms closely and consistently clustered around the diagonal. Thus, the error terms evinced a normal distribution.

An assessment of sample characteristics (Table 1) indicated that the majority of participants visited the GP 3–4 times a year and approximately a quarter had ME between 5 and 10 years. A majority of the sample were from the rest of the UK, female, and had received a formal diagnosis. However, approximately half of the sample reported that they did not trust the GP. Time to diagnosis suggested quite an even spread of the sample (apart from the ‘less than 3 months’ category which only a small fraction reported).

Chi-square tests of association assessed how each categorical predictor aligned with the number of GP visits (see Table 2). Results indicated a significant association between number of GP visits with length of time with ME, location, trust in GP, and gender. For length of time with ME, there appeared to be a general trend supporting the notion that the longer participants suffered from ME, the less frequently they visited the GP. For example, 8.2 and 43.3% of those in the highest category (20 years plus) visited monthly and 1–2 times a year respectively, whereas 25 and 12.5% of those in lowest category (1–2 years) visited monthly and 1–2 times a year respectively. Analysis of location suggested that a greater percentage of those in Greater Manchester regions visited their GP more frequently (52.5% visited more than 5 times a year), whereas 64.1% from the rest of the UK visited the GP less than 4 times a year. An evident difference existed concerning trust in GP; 35.3% of participants who did not trust the GP visited less than 4 times a year compared with 17.6% who trusted the GP, whereas 11.6% who did not trust the GP visited monthly compared with 21% of those who trusted the GP. Analysis of gender indicated that women (40.3%) were more likely to visit their GP more than 5 times a year compared with men (23.1%).

**Table 2:** Associations between predictor variables and number of GP visits

		Number of GP visits a year						
		1–2 (*n* = 134)	3–4 (*n* = 161)	5–6 (*n* = 113)	Monthly (*n* = 68)		Test of association with GP visits	
Variable	Category	*n* (%)	*n* (%)	*n* (%)	*n* (%)	Total *n*	*χ*^*2*^ test	*p* value
Time with ME	1–2 years	4 (12.5)	12 (37.5)	8 (25)	8 (25)	32		
	2–5 years	14 (20.6)	25 (36.8)	19 (27.9)	10 (14.7)	68		
	5–10 years	34 (28.3)	34 (28.3)	32 (26.7)	20 (16.7)	120		
	10–15 years	17 (21)	27 (33.3)	25 (30.9)	12 (14.8)	81		
	15–20 years	23 (29.5)	31 (39.7)	14 (17.9)	10 (12.8)	78		
	> 20 years	42 (43.3)	32 (33)	15 (15.5)	8 (8.2)	97		
	Total time with ME						28.062	.021*
Location	Rest of UK	125 (30)	142 (34.1)	94 (22.5)	56 (13.4)	417		
	Manchester borough	9 (15.3)	19 (32.2)	19 (32.2)	12 (20.3)	59		
	Total location						7.791	.050*
Trust in GP	Yes	21 (17.6)	43 (36.1)	30 (25.2)	25 (21)	119		
	No	79 (35.3)	67 (29.9)	52 (23.2)	26 (11.6)	224		
	Unsure	34 (25.6)	51 (38.3)	31 (23.3)	17 (12.8)	133		
	Total trust in GP						16.261	.012*
Formal diagnosis	Yes	129 (27.9)	157 (33.9)	111 (24)	66 (14.3)	463		
	No	5 (38.5)	4 (30.8)	2 (15.4)	2 (15.4)	13		
	Total diagnosis						.946	.814
Time to diagnosis	< 3 months	7 (25)	13 (46.4)	5 (17.9)	3 (10.7)	28		
	3–6 months	25 (32.9)	31 (40.8)	15 (19.7)	5 (6.6)	76		
	6 mths-1 year	31 (26.1)	44 (37)	22 (18.5)	22 (18.5)	119		
	1–2 years	25 (32.5)	21 (27.3)	22 (28.6)	9 (11.7)	77		
	2–5 years	22 (28.2)	19 (24.4)	21 (26.9)	16 (20.5)	78		
	> 5 years	24 (24.5)	33 (33.7)	28 (28.6)	13 (13.3)	98		
	Total time to diagnosis						19.458	.194
Gender	Female	106 (25.8)	139 (33.8)	105 (25.5)	61 (14.8)	411		
	Male	28 (43.1)	22 (33.8)	8 (12.3)	7 (10.8)	65		
	Total gender						10.753	.013*

*Note.* * indicates *p* < .05

A multinomial logistic regression evaluated the prediction of membership into GP visit categories (1–2 times a year, 3–4 times a year, 5–6 times a year, monthly). The reference group was 1–2 times a year. Analyses revealed a good model fit (discrimination among groups) on the basis of length of time with ME, location, trust in GP, formal diagnosis, time to diagnosis of ME, and gender, *χ*^*2*^ (483, *N* = 476) = 495.140, *p* = .341 (using deviance criterion), Nagelkerke *R*^*2*^ = .183. Similarly, a test of the full model vs. the constant model revealed a significant result, *χ*^*2*^ (45, *N* = 476) = 88.760, *p* < .001, suggesting that the predictors as a group satisfactorily distinguished between the GP visits categories.

The Wald statistic (see Table 3) indicated that compared to individuals who visited the GP 1–2 times a year, individuals who visited the GP 3–4 times a year were significantly more likely to have suffered from ME for 2–5 years (OR = 2.695) and less likely to be from a Greater Manchester borough (OR = .411). Individuals who visited the GP 5–6 times a year were significantly more likely to have had ME between 1 and 15 years (1–2 years: OR = 8.771; 2–5 years: OR = 5.369; 5–10 years: OR = 3.180; 10–15 years = 4.310) and to be female (OR = 3.686). Individuals visiting 5–6 times a year were also less likely to be from a Greater Manchester borough (OR = .321) and for time to diagnosis to be 3–6 months (OR = .328) compared to the reference group. Lastly, individuals who visited the GP monthly were significantly more likely to have had ME between 1 and 15 years (1–2 years, OR = 11.632; 2–5 years: OR = 4.633; 5–10 years: OR = 3.397; 10–15 years: OR = 3.372), to trust the GP (OR = 2.503), and to be female (OR = 2.849). Individuals visiting monthly were furthermore less likely to be from a Greater Manchester borough (OR = .247) and for time to diagnosis to be 3–6 months (OR = .205).

**Table 3:**
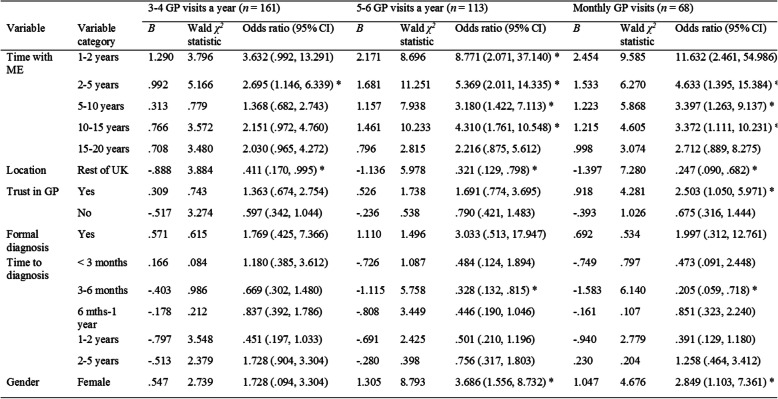
Multinomial logistic regression differentiating 1–2 GP visit a year (*n* = 134) from 3 to 4 GP visits, 5–6 GP visits and monthly GP visits

Note: References categories: length of time with ME = more than 20 years, location = Manchester borough, trust in GP = unsure, formal diagnosis of ME = no, time to diagnose of ME = more than 5 years, genders = male; * indicates *p < .05*

## Discussion

A significant proportion of ME suffers reported unsatisfactory relationships with their GP. This finding indicated that ME patients experience a troubled relationship with their primary health contact. Indeed, approximately half of the surveyed participants did not trust their GP. Trust was only evident within the monthly GP visit group. These are important outcomes because lack of trust can negatively affect the number of times patients visit GPs and condition management [17, 21, 27]. One strategy with the potential to address this issue is for GPs to cultivate a sense of “we-ness”. Shared GP/ME patient group membership and common goals may facilitate respect and trust [4, 24]. Building rapport with this cohort is vital.

Potential barriers to positive in-group inclination are depersonalization and stereotyping. From the perspective of GPs, the ME patient group are problematic because ME diagnosis is often considered contentious [15, 24], prevalence is low, and, importantly, ME is absent from the Quality and Outcome Framework [3]. These factors combine to make ME management and treatment challenging and time consuming [15, 16, 20]. Within the present study, formal diagnosis and time to diagnosis had little effect on ME patient visits. The notable exception was the 3–6 month category, where diagnosis was associated with increased GP visits in the 5–6 a year and monthly categories. These results suggest this is an important period for diagnosis. Subsequent research should examine this further.

Moreover, the groups that patients understand themselves as belonging to potentially influences symptom appraisal/responses, health related norms/behaviours, coping, social support and clinical outcomes [22, 23]. Thus, from a health and well-being perspective, it is clear that individual’s relationships, and identification, with their medical and social care providers are of vital importance. Indeed, for people living with chronic conditions, the GP relationship is a crucial factor [4, 18, 19]. As such, the functionality of the doctor patient interaction is fundamental to individual well-being. Mutual respect is a core facet at the heart of this relationship [3, 17].

Illustratively, St Claire and Clucas [27] observed that patients reported several affirmative outcomes when they perceived their doctor as respectful (i.e., greater satisfaction, intention to adhere to advice, and inclination to revisit). Thus, positive patient regard not only enhances the doctor and patient interaction, but also improves patient prognosis by reducing symptoms and facilitating healthy behaviours. In this context, communication and skills training may help to enhance GP interaction with ME patients. Succeeding studies should assess this area.

The results of the present study also revealed a negative relationship between length of ME and frequency of GP visits, as the length of the condition extended patients reported fewer GP visits. One way to understand this is to build conceptually on a recognition of the importance of communication. Consistent with the positive link between trust and GP visits [3, 17], De Carvalho Leite et al. [28] reported that inadequate communication between patients and professionals was often a barrier to care. With a significant proportion of English GPs reported as being sceptical of ME as a diagnosis [3, 14], it is not surprising that patients frequently report their attempts to access services as ‘exhausting, demoralising and isolating’ [15, 20, 28].

In line with this qualitative literature, our findings reveal that over time ME patients engage less with medical practitioners. Although, the reasons for this are currently unclear, this finding suggests that improved doctor and ME patient communication and monitoring is advisable. This would help to track number of appointments attended, frequency, and points at which attendance cease. Maintaining and engaging with treatment is vital to condition management and well-being. A general remedy for ineffective communication is to provide empathic, personalised and co-ordinated support from health and social services [3, 17, 28]. Additionally, when ME patients stop visiting their GP it is recommended that reasons for non-attendance are sought. Collation and consideration of such feedback could usefully inform subsequent investigation.

This study found also that gender was an important factor. Specifically, women were more likely to visit their GP than men. Indeed, analysis of gender revealed that women were more likely to visit their GP more than 5 times a year (40.3%) compared with men (23.1%). Psychology has long recognised the importance of gender [6, 29, 30] which is most usefully considered as a function of social and developmental factors rather than as an essentialist actuality. Sandberg, Pasterski, and Callens, [31] for example argued that different psychosexual developmental experiences manifest in women as a sense of self that is relational, whereas men’s sense of self is more independent and less contingent on interpersonal connection [5, 23, 30].

Gender differences in health service access have received relatively little attention in the UK. When studies evaluate gender, they focus typically on women’s issues, particularly male privilege [6, 32]. The present article highlighted the fact that men also possess important healthcare needs. Hence, effective provision needs to recognise the needs of all gender groups. Haslam [33] argues that, from clinical and health perspectives, in order to engage usefully with identity (including gender) researchers need to work with an individual’s sense of self rather than across it. Study findings recommend that health care professionals would benefit from increased awareness of this issue.

One mechanism through which gender differences may arise is stereotyping. Stereotyping defines appropriate and relevant behaviours within particularly contexts and guides expectations [30, 34, 35]. For example, the typical male stereotype implies that a person has psychological capability, goal-orientation, self-confidence as well as social dominance [30, 35]. This implies that men are resilient [34]. Moreover, the male stereotype positions ‘real’ men as being invulnerable [29, 35].

Importantly this process of categorization links to existing social relations, [22, 23, 36], including relationships with medical service providers [4, 6]. The norms of stoicism and control associated with masculinity often prevent men from seeking healthcare where they believe they risk lapsing into a passive and/or ‘feminine’ sick role that is associated with weakness [32]. The argument presented in this paper, driven by the finding that men are less likely to be engaged with their GP, is that medical practitioners need to engage with men, recognize and harness these male identities/stereotypes and norms by working with, rather than across them [4, 22, 33].

A final important variable was geographic location. Isakson et al. [37] contend that when there are preventable health inequities between people, the distribution of health resources that shape the inequity require consideration. Such questioning is the raison d’être of Healthwatch networks. Indeed, Healthwatch Trafford commissioned this patient experience gathering exercise because of reported patient issues within the Greater Manchester region. Specifically, they were keen to determine whether there were variances in ME treatment compared with the rest of the UK. Results revealed a significant difference. Greater Manchester residents living with ME visited their GPs more frequently than those living in the rest of the country did. Future research should attempt to unpack this finding in order to identify what this might be.

## Conclusions

Overall, study findings should usefully inform a range of salient and potentially crucial social relationships and interactions that affect the lives of those living with ME. Significant groups include the clinical dyad of GP and patient, gendered groups (i.e. men and women), the patient group (i.e. those living with ME), and the position of those living with ME vis-a-vis wider society (e.g. employers, social welfare, etc.). Haslam [33] argues powerfully that to harness the power of relationships we need to work with the understandings that people have of themselves. The patient experiences presented in this paper suggests that there is ample scope in the field of ME for medical practitioners to harness that advice to their patient’s advantage.

## Data Availability

The data used in the current study is available in the MMU repository and also available directly, on request, from the corresponding author.

## Abbreviations

ME: Myalgic Encephalomyelitis
GP: General Practitioner
CDC: Centres for Disease Control and Prevention
CFS: Chronic fatigue syndrome
